# Genetic consequences of pond production of a pikeperch (*Sander lucioperca* L.) stock with natural origin: the effects of changed selection pressure and reduced population size

**DOI:** 10.7717/peerj.8745

**Published:** 2020-03-17

**Authors:** Tamás Molnár, Ildikó Benedek, Balázs Kovács, Attila Zsolnai, István Lehoczky

**Affiliations:** 1 Institute of Environmental Sciences and Nature Conservation, Kaposvár University, Kaposvár, Hungary; 2 Department of Aquaculture, Szent István University, Gödöllő, Hungary; 3 Institute for Farm Animal Gene Conservation, National Centre for Biodiversity and Gene Conservation, Gödöllő, Hungary; 4 Research Institute for Animal Breeding, Nutrition and Meat Science (ATHK), National Agricultural Research and Innovation Centre, Herceghalom, Hungary

**Keywords:** *Sander lucioperca*, Pond culture, Diversity loss, Genetic selection, Microsatellite, Ryman–Laikre effect, Effective population size

## Abstract

The pikeperch (*Sander lucioperca* L.) possesses great potential for diversifying European aquaculture. However, studies on the genetic risk of stocking natural waters with farmed individuals of this species have been limited. Even the effect of pond culture on the genetic composition of stocks with natural-origin has not yet been determined. Our study aimed to compare the genetic variability of a wild living pikeperch population, a pond cultured broodstock (originating from the wild population) and its offspring generation. We also aimed to detect the potential signs of selection using three different methods. By analyzing the molecular data with 14 microsatellite markers, we illustrated that the impact of pond culture on the genetic diversity of fish stocks is similar to hatchery rearing due to its diversity reducing effect caused by using lower effective population sizes. Although the heterozygosity was similar in all populations (H_o_ = 0.68–0.71), the average number of alleles and allelic richness were significantly lower in the pond cultured stocks (NA = 7.5 and 6; AR = 7.5 and 5.9) compared to the wild population (NA = 11.00, AR = 10.47). Despite the semi-natural conditions of the present study, we detected changing selection pressure in one of the 14 microsatellite markers.

## Introduction

Stock enhancement of cultured species usually aims to compensate for exploitation shortages and to foster fisheries (Guillerault et al., 2018). Besides, that stock enhancement can pose a risk even at the ecosystem level (since economically valuable species are often at the top of the food chain and significantly affect the other species in the chain) it has significant effect on the genetic structure of the enhanced stock (Grant et al., 2017). The ideal method for stocking natural waters with artificially propagated fish should be based on the common origin of the natural population and the artificially propagated broodstock. The genetic variability of the broodstock used for propagation must represent the genetic diversity of the original population (Arlinghaus et al., 2015). Hybridization of individuals from aquaculture stocks and wild populations presents different genetic risks: (1) the loss of genetic variation within and among populations, (2) the loss of adaptations (reduction of fitness), (3) change in population composition and (4) change in population structure (Laikre et al., 2010, Waples et al., 2016).

Supplementation programs have mainly focused on populations with low effective population sizes and/or low genetic diversity (Fraser et al., 2019). Any type of supplementation program can affect the genetic composition of the target population due to several factors. The first factor is the reduced effective population size of the hatchery stock (as a consequence of the small number of individuals used as broodstock). This factor could result in the genetic degradation of the wild population even in one generation, depending on the varying reproductive success of the supplemented individuals (Christie et al., 2012a). The potential reducing effect of supplementation on effective population size of wild populations is presented in the Ryman–Laikre model (Ryman & Laikre, 1991). This model shows that the effective population size (Ne) of the supplemented population depends on the Ne of the captive and wild populations and the fraction of artificially produced progeny (x). The extremely small captive Ne and large x could result in an even lower final Ne than that without population supplementation. Waples et al. (2016) improved this model by highlighting the importance of the numbers of captive and wild adults (N), the ratio of Ne/N in captive and wild stocks and that the capture of individuals for supportive (captive) breeding lowers the Ne of the wild population.

Beside captive broodstock size, the other main factor is the fitness of captive individuals in the wild. The genetic diversity of wild populations enables them to respond to both natural (ecological) and anthropogenic challenges (Grant et al., 2017), resulting in better tolerance in larger populations under culture conditions. Due to local adaptations of the wild variants, the differences in the characteristics of captive bred individuals often result in poor adaptation when introduced into the wild as part of species recovery programs. A captive-breeding prefers traits that are adaptive to the culture environment but also generate maladaptive phenotypes after released into the nature (Fraser et al., 2019). One of the key parameters in evaluating fitness is relative reproductive success (RRS). In salmonids, significant differences have been observed between fitness in captivity and fitness in the wild. Hatchery adults’ reproductive success in the wild is always lower than that of wild adults and there could be differences in RRS according to sex (the male hatchery fish RRS is lower than female hatchery fish RRS) (Christie, Ford & Blouin, 2014). The proportional contributions of hatchery-reared individuals in marine species range between 0.01 and 0.3 (Grant et al., 2017).

The effects of population supplementation programs have been described in several aquaculture (mainly salmonid and marine) species (reviewed by Araki & Schmid (2010), Arlinghaus et al. (2015), Grant et al. (2017)). However, in the past few decades, the diversification of aquaculture has been focused on new species. The pikeperch (*Sander lucioperca* L.) exhibits great potential for the diversification of European aquaculture (FAO, 2017). The control of reproduction and the bio-economical suitability and sustainability of intensive pikeperch breeding has been established in several studies. The demand for this good quality fish has increased along with the decline of natural water catches in Russia, Finland and Estonia (from 50,000 metric tons in the 1950s to 20,000 tons currently). At the same time, aquaculture production has increased to roughly 900 tons in the last decade from 50 tons in 1950. Generally, wild and cultured 2–6 year old broodfish are used for induced propagation or natural/semi natural spawning in order to produce larvae for the stocking of fishponds and natural waters (FAO, 2017).

In a commercial cultured species like pikeperch, the efficiency of production and future breeding programs is largely influenced by the genetic variance of the species. The basis of this variance is the genetic diversity of the native populations. The genetic diversity of the species was described in Aral-Lake (Khurshut & Kohlmann, 2009) and the Caspian Sea (Gharibkhani et al., 2009) and in Kazak (Barmintseva et al., 2014), French (Poulet et al., 2009; Louati et al., 2016), German (Eschbach et al., 2014), Fennoscandian (Björklund, Aho & Larsson, 2007; Säisä et al., 2010; Salminen et al., 2012), Russian (Kusishchin et al., 2018) and Hungarian (Kánainé Sipos et al., 2019) populations.

This euryoecious species has the ability to use a broad range of environmental conditions and the native populations adapt to their local conditions. Eschbach et al. (2014) compared German populations in the native and non-native range of the species and found that the non-native genotypes had much less influence on the genetic background of native populations.

Salminen et al. (2012) described the genetic changes in three indigenous populations after stocking non-native individuals. The three populations showed different patterns: the genetic structure of the first one was almost identical with the original, the original genetic background of the second population was nearly extinct and the third was diluted by the stockings. These results show that the RRS in pike perch can vary to a great extent, that genetically adapted native populations can be competitively superior to the stocked (maladapted under culture conditions) conspecifics and that the enhancement of self-sustaining populations is rarely successful. On the other hand, these results highlight the demand for further studies on the genetic background of stocking material.

Hungarian stock has a unique genetic background as it segregates from the Baltic population and differs from stock found near Central Europe. This suggests the presence of a third group associated with Hungarian lakes probably dispersed throughout the Danube River (Tsigenopoulos et al., 2018). Lake Balaton is the largest shallow lake (surface area: 593 km^2^; mean depth: 3.2 m) in Central-Eastern Europe, where the native pikeperch is the main piscivorous species. Although commercial fishing has been banned there since 2014, the maintenance of the pikeperch population requires regular stocking due to the high rate of angler catches. Until recently, 1–1.5 million of 3–5 cm length fingerling produced in fish ponds were stocked annually in the lake (Specziár & Turcsányi, 2017). These fish ponds represent natural or semi natural conditions, as food availability and predation are the most important differences compared to Lake Balaton (Tölg, 1984). The environmental alterations could have a selective influence on the genetic background amplified by the management practices during production.

Our study aimed to compare the genetic variability of a wild living pikeperch population (Lake Balaton), a pond cultured broodstock (originating from the wild population) and its offspring generation. Our objectives were: (i) to describe the genetic patterns (genetic diversity and genetic structure) of the different stocks; (ii) to outline the causes that may have driven genetic patterns in pikeperch (founder effect, genetic drift and selection); and (iii) to analyze how current hatchery practices might affect the management of natural populations. The broodstock had been maintained in fish ponds and the offspring generation was propagated artificially by a company that dominates the pikeperch larvae and fingerling market in Hungary.

## Materials and Methods

### Sample collection and DNA preparation

This study was approved by the Committee on the Ethics of Animal Experiments of the Kaposvár University (permit number: 3/2016-MÁB). Field experiments were approved by the Hungarian Ministry of Agriculture (permit number: HHgF/269-1/2015). Fin samples of wild fish were collected from Lake Balaton (a shallow, oligo-mesotrophic lake with a surface area of 596 km^2^ and a mean water depth of about 3 m, located in Hungary, Central-Eastern Europe) from anglers’ catchings (from six localities: Keszthely, Tihany, Balatonfüred, Balatonboglár, Balatonakali and Siófok, total sample number *n* = 46) in the period of 2016–2017. BoFa Fish Farm (located in Attala, South-West Hungary) provided samples of the hatchery stocks (broodstock *n* = 40 and offspring *n* = 44) in the 2016 breeding season. The broodstock had been maintained with artificial propagation since 2005. The stock included approximately 70–80 individuals from which 40–50 individuals were annually selected for propagation (based on morphological characteristics). The other broodfish were replaced with individuals bred on the farm, resulting in overlapping generations in the broodstock. The generation time of the species was 4 years. In the experiment, all propagated brood fish were sampled and used to produce fingerlings (an “offspring” sample). We took precautions to handle the live fish in a way that minimized possible stress for them. Fin samples were collected in 96% ethanol and stored in a deep freezer (−80 °C) until ready for processing. The genomic DNA was extracted with DNeasy Blood and Tissue kits (Qiagen, Hilden, Germany) following the extraction protocol of the manufacturer. DNA quality and quantity were checked using the Maestro Nano Drop Spectrophotometer MN-913 (MaestroGen, Taiwan, China). The sample DNA concentration was equalized to 50 µg/ml.

### Microsatellite analysis

A total of 15 microsatellite DNA markers (MSL1, MSL2, MSL3, MSL5, MSL6, MSL9—Kohlmann & Kersten (2008); Svi-4, Svi-6, Svi-L7, Svi-L8, Svi-18—Wirth, Saint-Laurent & Bernatchez (1999); Pfla-L3, Pfla-L8—Leclerc, Wirth & Bernatchez (2000), Za138, Za199—Dubut et al. (2010)) were used to genotype all individuals. The markers were amplified in three multiplex PCRs using NED, PET, VIC and FAM end-labeled primers (Table 1).

**Table 1:** Primers used in three multiplex reactions (A, B, C) to genotype the listed loci in pikeperch populations.

Locus	GenBank Acc. no.	Multiplex PCR	Primer concentration multiplex PCR (µM)	Primer sequence
MSL-1	EF694018	A	0.1	F-NED-TGTTTGTCAGCGTCAAGAGG
				R-TTCCGCTCCAACATATCACA
MSL-3	EF694020	A	0.066	F-NED-CCGGCATCCATACACCTTAC
				R-CACACCTGTGTCTGCCTAACA
MSL-5	EF694022	A	0.266	F-PET-CAATCGCTCTGAGGATGTCA
				R-AAGGGTGGGGAAATTATTCG
MSL-6	EF694023	A	0.2	F-FAM-GTCGTCATCGTCAGCACAGT
				R-ACTACACGGGACGCTGGA
MSL-9	EF694026	A	0.2	F-VIC-GCATCACTTGCGTCACTTTC
				R-GCAGTCAGTGCTTGAAGTGG
MSL-2	EF694019	B	0.2	F-PET-TTTTCACACCGTGCATGACT
				R-ACCCTCAGCCTCTGTGTACG
Pfla L8	AF211833	B	0.2	F-FAM-GCCTTATTGTGTGACTTATCG
				R-GGATCTTTCACTTTTTCTTTCAG
Svi18	G36964	B	0.2	F-PET-GATCTGTAAACTCCAGCGTG
				R-CTTAAGCTGCTCAGCATCCAGG
Svi4	G36961	B	0.1	F-PET-ACAAATGCGGGCTGCTGTTC
				R-GATCGCGGCACAGATGTATTG
Svi 6	G36962	B	0.1	F-NED-CATATTATGTAGAGTGCAGACCC
				R-TGAGCTTCACCTCATATTCC
Svi-L7	AF144740	B	0.2	F-NED-GATGTGCATACATTTACTCC
				R-GCTTTAATCTGCTGAGAAC
PflaL3	AF211828	C	0.1	F-FAM-GCCGAATGTGATTGAATG
				R-CGCTAAAGCCAACTTAATG
Za138	HM622317	C	0.25	F-VIC-TTCTTTATACAAGAGGAATAGTTGCAG
				R-TTTTTGTGATTGTGCTATTTTAAAGG
Za199	HM622334	C	0.05	F-NED-CCTTCCCCTCAAAAGCATGT
				R-AGGAAATGGAAAGGGAATGC
SviL8	AF144741	C	0.3	F-PET-GCTTATACGTCGTTCTTATG
				R-ATGGAGAAGCAAGTTGAG

Amplifications were carried out in 20 µl reaction volume and polymerase chain reaction (PCR) was conducted using AmpliTaq Gold^®^ DNA Polymerase (Promega Corporation, Madison, WI, USA) with Buffer II (100 mM Tris–HCl, pH 8.3, 500 mM KCl). The final reaction conditions and temperature profiles of the multiplex reactions are presented in Table S2.

The PCR was carried out in a Px2 Thermal Cycler (Thermo Electron, Waltham, MA, USA). The products of the multiplex PCR reactions were pooled together and run against a Genescan 600 LIZ internal size standard (Applied Biosystems, Foster City, CA, USA) on a 3,500 Genetic Analyzer (Applied Biosystems, Foster City, CA, USA). After electrophoretic runs, PCR fragments were sized using GeneMapper version 4.0 software (Applied Biosystems, Foster City, CA, USA).

### Statistical analyses

The evidence for the presence of null alleles at each locus and correction (Monte Carlo simulation (bootstrap) method) was evaluated using MICRO-CHECKER version 2.2.3 (Van Oosterhout et al., 2004). The number of randomizations was 1,000 and a 95% confidence interval was used in the analysis. The number of alleles, observed and unbiased expected heterozygosity and F_IS_ were calculated using GenAlEx 6.5 software (Peakall & Smouse, 2012). We estimated allelic richness and private allelic richness using the rarefaction procedure with HP-RARE 1.0 software (Kalinowski, 2005). The H_o_ and F_IS_ values were standardized for population sizes using weighted means between the populations. The comparison of the genetic variability indices was performed using a Mann–Whitney *U*-test with the Bonferroni correction (a significance level of 0.016) (SPSS for Windows 11.5). We performed examinations of observed and expected heterozygosity over all loci, deviations from the Hardy–Weinberg equilibrium (HWE) using a Markov chain exact test (dememorization number: 5000, number of batches: 500, number of iterations per batch: 5,000) (Guo & Thompson, 1992) for each locus in each population, tests for linkage disequilibrium for each pair of loci in each population and tests for each locus and population using GENEPOP on the web (Raymond & Rousset, 1995, Rousset, 2008). Individual levels of multi-locus heterozygosity (attained by scoring individuals as heterozygous—1 or homozygous—0 and averaging across all loci (Blanchet et al., 2008)), were also compared between the three groups using a Mann–Whitney *U* test with a Bonferroni correction (a significance level of 0.016).

GenAlEx 6.5 software was used to perform Analysis of Molecular Variance (AMOVA) and to calculate pairwise Fst values and their significance (9,999 permutations were used for testing statistical significance).

The STRUCTURE software (Pritchard, Stephens & Donnelly, 2000; Falush, Stephens & Pritchard, 2003) was used to infer population structure. For estimating *K*, the most probable cluster number, posterior probabilities are calculated. The software uses a Bayesian clustering approach applying Markov Chain Monte Carlo (MCMC) estimation. For assessing the number of population clusters, an admixture scenario with allele frequencies correlated was chosen, the burn-in was set to 10^4^ and the number of further MCMC ran to 10^5^. Calculations were repeated 10 times for each *K*. The Δ*K* (a quantity based on the second order rate of change with respect to *K* of the likelihood function), was calculated using the STRUCTURE HARVESTER software (Earl & VonHoldt, 2012), a method by Evanno, Regnaut & Goudet (2005).

A UPGMA tree was constructed based on the codominant genotypic distances (calculated using GenAlEx 6.5 software) with MEGA6.06 software (Tamura et al., 2013). Relatedness between broodstock and offspring individuals was calculated with ML-Relate using 0.95 confidence interval (Kalinowski, Wagner & Taper, 2006).

Discriminant Analysis of Principal Components (DAPC) using microsatellite loci and populations was performed in R environment (3.4.0) with ADEGENET 2.0.1. package (Jombart, 2008) to partition the variance into a between-group and within-group component, in an effort to maximize discrimination between the three groups (Broodstock, Offspring, Wild).

The effective population size (Ne) of the stocks was calculated in NeEstimator 2.1 (Do et al., 2014) using the linkage disequilibrium method (Bartley et al., 1992) for all three populations and the temporal method according to broodstock and offspring populations (Jorde & Ryman, 2007). The threshold for the exclusion of rare alleles was 0.01. We also tested for recent and major reductions in population size using BOTTLENECK 1.2.02 (Piry, Luikart & Cornuet, 1999). Significance was tested with the Wilcoxon sign-rank test, under a two-phase mutation model (TPM), 95% single-step mutations and 5% multiple-step mutations (and a variance among multiple steps of approximately 12) for 5,000 iterations as recommended by Piry, Luikart & Cornuet (1999).

Loci under directional selection were expected to have lower intrapopulation variability and larger interpopulation variability than neutral loci. Three methods were used for detecting the sign of selection: LOSITAN (Antao et al., 2008), BayeScan v2.01 (Foll & Gaggiotti, 2008) and lnRH (Kauer, Dieringer & Schlotterer, 2003). The comparison of several outlier detection methods showed that these methods differed in the number of false positives and false negatives (Narum & Hess, 2011), therefore loci determined in at least two methods were scored as a putative outlier. LOSITAN is based on an island model that uses a coalescent Fst-outlier method based on the distribution of Fst as a function of heterozygosity. A stepwise mutation model with 0.99 confidence interval, 0.1 false discovery rate and 10^6^ simulations was used. BayeScan uses a logistic regression model that explains the observed pattern of diversity by dividing it in a locus- and population-specific component (Beaumont & Balding, 2004). We used standard parameter settings with 20 pilot runs of 5,000 iterations and a burn-in of 50,000 iterations. We considered loci as outliers if the hypothesis of selection was supported by log_10_ (PO) > 1.5. The third method, the lnRH method, could separate selective sweeps from demographic effects, such as bottlenecks. The lnRH test calculates the logarithm to the ratio of *H* for each locus for a pair of populations. *H* is defined from the expected heterozygosity by the following equation: $[1/(1-{\rm He})]^{2}$ (Kauer, Dieringer & Schlotterer, 2003). The lnRH values of neutral loci are normally distributed and loci that are outliers (with 95% limit) in the distribution are candidates for selection.

As the genome sequence for pikeperch is not available in the GenBank database, the putative outlier loci were blasted against the NCBI BLAST Transcriptome Shotgun Assembly (TSA) database, limited by the organism *Perca fluviatilis* (taxid: 8168) using the program discontiguous megablast with default settings. The contigs above an “*E*-value” significance threshold of 1 × 10^−4^ were further blasted against NCBI BLAST nucleotide collection database using the program megablast with default settings.

## Results

### Genetic diversity

The Microchecker did not detect evidence for large allelic dropout and the presence of null alleles was assumed only in the case of loci PflaL8 (in all populations) and SviL8 (in the broodstock population) due to general excess of homozygotes. PflaL8 locus was removed from the downstream analyses, but SviL8 was retained as its frequency was low (6%). Basic molecular genetic parameters are shown in Table 2.

**Table 2:** Basic molecular genetic parameters of the populations under study. N, number of animals; uH_e_, unbiased expected heterozygosity; H_o_, observed heterozygosity values; *N*_A_, average allele numbers; AR, allelic richness; AR_p_, private allelic richness; *F*_IS_, inbreeding coefficient. If indicated, “a” and “b” upper case letters indicate significant (*p* < 0.05) differences among the groups.

Population	*N*	uH_e_	*H*_o_	*F*_IS_	*N*_A_	AR	AR_P_
Wild	46	0.70 ± 0.12	0.68 ± 0.16	0.020 ± 0.08	11.00 ± 4.26^a^	10.47 ± 3.98^a^	3.81 ± 2.01^a^
Broodstock	40	0.72 ± 0.10	0.71 ± 0.10	−0.010 ± 0.10	7.50 ± 3.34^b^	7.50 ± 3.34^b^	0.43 ± 0.75^b^
Offspring	44	0.68 ± 0.11	0.70 ± 0.16	−0.036 ± 0.11	6.00 ± 1.46^b^	5.9 ± 1.47^b^	0, 0 ± 0, 0^c^

The diversity decreased in the broodstock and the offspring when compared to the wild population, meeting our expectations. The offspring showed even lower diversity than the broodstock but the difference was significant only in private allelic richness. The unbiased expected heterozygosity (uHe) values were high (ranging between 0.68 and 0.72) and there were no significant differences between the three examined groups (effect size of difference (d) was 0.36, 0.18 and −0.18 between broodstock-offspring, broodstock-wild and offspring-wild groups, respectively). The observed heterozygosity values (H_o_) were also high (ranging between 0.68 and 0.71) and the differences between the three groups were also not significant (*d* = 0.07, 0.27 and 0.18 between broodstock-offspring, broodstock-wild and offspring-wild groups, respectively). The average allele numbers (NA) per loci ranged between 6.00 and 11.00. The differences between wild and broodstock (*d* = 0.92, *Z* = −2.740, *P* = 0.006) and wild and offspring populations (*d* = 1.31, *Z* = −3.766, *P* = 0.001) were significant. The allelic richness (AR) data showed significant differences between all groups, but after the Bonferroni correction, only wild-broodstock (*d* = 0.82, *Z* = −2.446, *P* = 0.014) and wild-offspring (*d* = 1.27, *Z* = −3.493, *P* = 0.001) comparisons remained significant and broodstock and offspring were not different (*d* = 0.43, *Z* = −1.971, *P* = 0.049). In the case of private allelic richness (ARp) all populations differed significantly (wild-broodstock *d* = 1.60, *Z* = −3.888, *P* = 0.001; wild-offspring *d* = 1.81, *Z* = −4.545, *P* = 0.001 and broodstock-offsping *d* = 0.20, *Z* = −4.015, *P* = 0.001). The offspring population showed no private alleles while the average ARp was 0.43 in the broodstock and 3.81 in the wild population. The following loci showed deviation from the HW equilibrium only in one population: PflaL3 in the broodstock population; MSL-1, MSL-5, MSL-6 and Svi-18 in the offspring population; and MSL-9 and MSL-3 in the wild population. F_IS_ values were generally low and in the negative range, except for the wild population, but the difference between them was not significant (*d* = 0.30, 0.50 and −0.20 in the wild-broodstock; wild-offspring and broodstock-offsping groups). The averages of individual levels of multilocus heterozygosity (Blanchet et al., 2008) were 9.67 (±1.49), 10.00 (±1.90) and 9.86 (±1.40), in the wild, broodstock and offspring groups, respectively. However, the groups did not differ significantly.

There is significant evidence to support the hypothesis that management can alter the effective population size. The values of the effective population sizes estimated by the linkage disequilibrium method were 1,032.6 (95% CI [306.5–infinite]), 62.5 (95% CI [47.9–86.6]) and 15.1 (95% CI [12.9–17.7]) individuals in the wild, broodstock and offspring populations, respectively. The temporal method according to broodstock and offspring populations found that Ne was 31.3 (95% CI [23.0–89.9]) individuals. Unexpectedly, results from the Wilcoxon sign-rank test for heterozygosity excess performed using BOTTLENECK suggested a recent population bottleneck only in the wild (Balaton) population (*P* = 0.00006). An excess of heterozygosity compared to expectations under drift-mutation equilibrium was calculated in all of the 14 loci against the expected value of 8.23 and all of them was significant.

### Genetic structure

The AMOVA results showed that only 7.87% of the variance was found among populations indicating low/moderate levels of population differentiation. This was confirmed by the significance (*P* < 0.05) of pairwise Fst values between population pairs. A low Fst value (0.011) was detected between broodstock and offspring while moderate values were found between wild and broodstock (0.089) and wild and offspring (0.123) populations.

**Figure 1:**
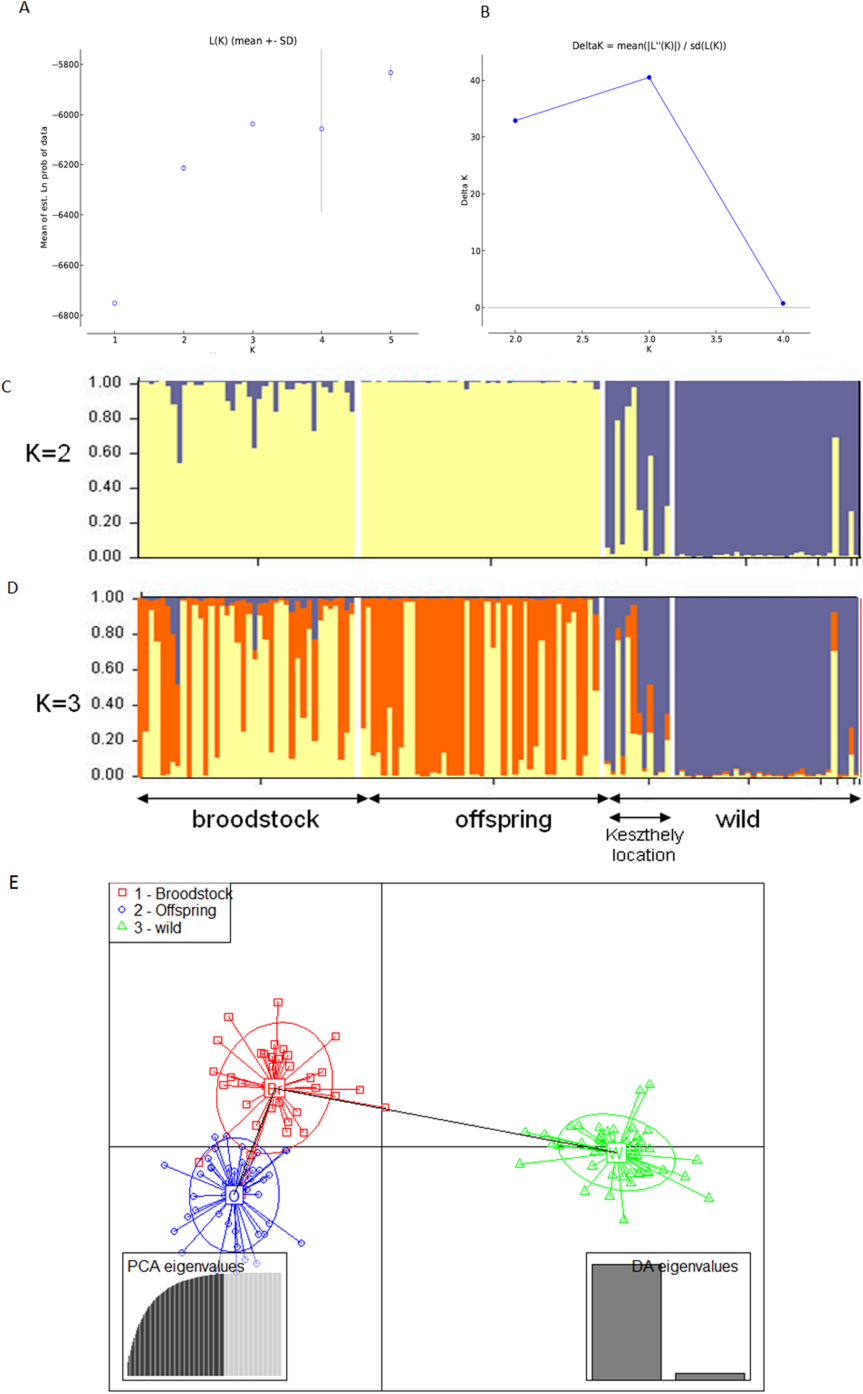
Bayesian STRUCTURE clustering results of microsatellite variation among the three populations using the full dataset (14 markers). (A) Estimation of *K*, posterior probabilities (highest lnP(D)) and (B) estimation of *K* the Δ*K* method of Evanno. The most probable cluster number is *K* = 3 (mean LnP(*K*) = −6,037.19; Δ*K* = 40.499). (C) Distribution of the three genetic clusters determined by STRUCTURE in the individuals of the examined populations (*K* = 2). (D) Distribution of the three genetic clusters determined by STRUCTURE in the individuals of the examined populations (*K* = 3) Population 1 (individuals 1–40) Broodstock, Attala population, Population 2 (Individuals 41–84) Offspring, Attala Population, Population 3 (individuals 85–130)-wild, Balaton population—individuals 85–96 represents a subpopulation in the Keszthely basin. (E) Representation of DAPC of individuals obtained by ADGENET. The axes represent the first two Linear Discriminants (LD), the *x* axis the fits to the first LD, while the *y* axis refer to the second LD. The Groups are displayed by different colors and inertia ellipses.

**Figure 2:**
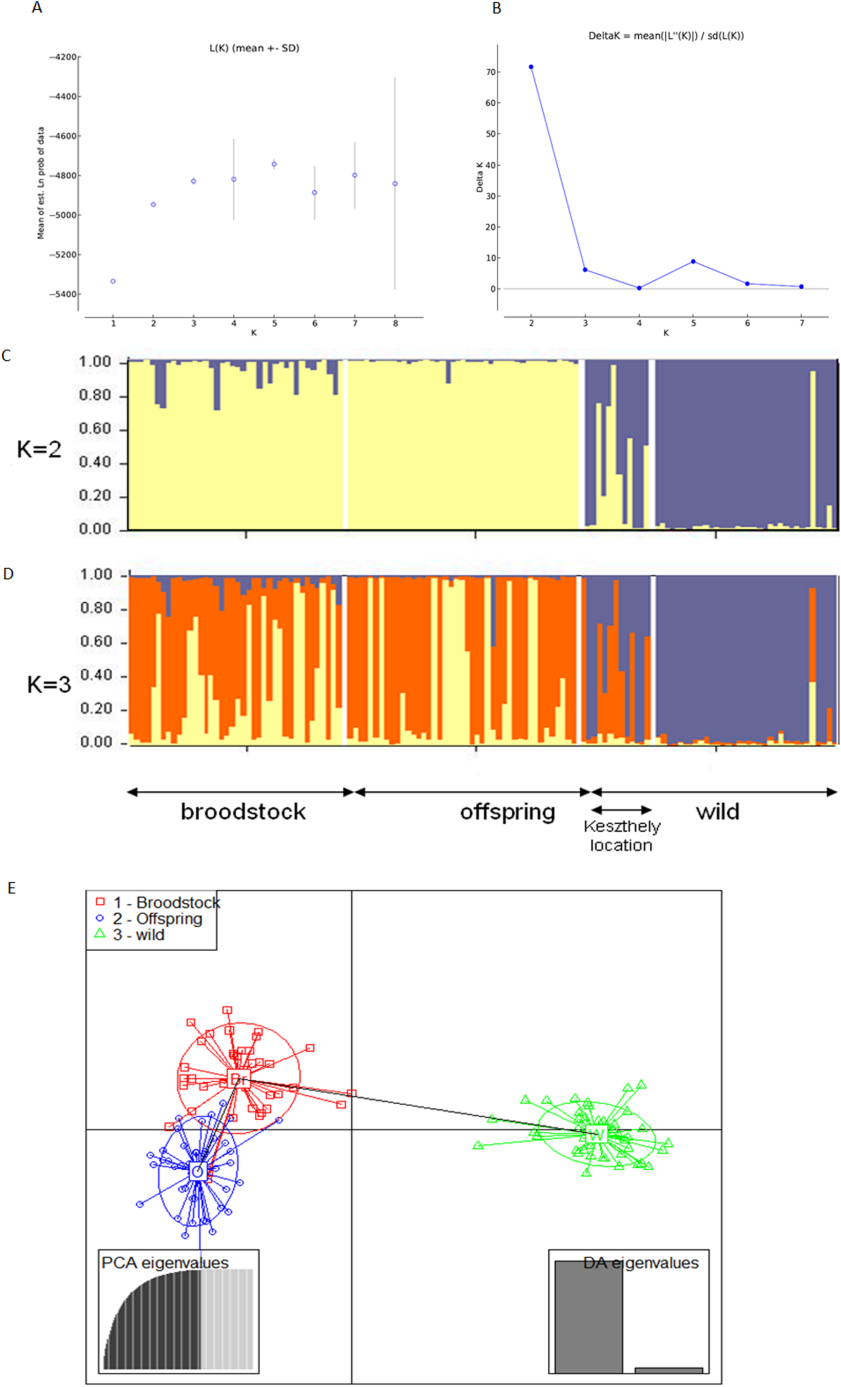
Bayesian STRUCTURE clustering results of microsatellite variation among the three populations using the neutral dataset (13 markers). (A) Estimation of *K*, posterior probabilities (highest lnP(D)) and (B) estimation of *K* the Δ*K* method of Evanno. The most probable cluster number is *K* = 3 (mean LnP(K) = −6,037.19; Δ*K* = 40.499). (C) Distribution of the three genetic clusters determined by STRUCTURE in the individuals of the examined populations (*K* = 2). (D) Distribution of the three genetic clusters determined by STRUCTURE in the individuals of the examined populations (*K* = 3) Population 1 (individuals 1–40) Broodstock, Attala population, Population 2 (Individuals 41–84) Offspring, Attala Population, Population 3 (individuals 85–130)-wild, Balaton population—individuals 85–96 represents a subpopulation in the Keszthely basin. (E) Representation of DAPC of individuals obtained by ADGENET. The axes represent the first two Linear Discriminants (LD), the *x* axis the fits to the first LD, while the *y* axis refer to the second LD. The Groups are displayed by different colors and inertia ellipses.

The existence of the three original populations was supported by structure analysis that found that the most probable cluster number was three (*K* = 3, mean LnP(K) = −6,037.19; Δ*K* = 40.499) using both neutral and outlier markers in the calculation (Fig. 1). If only neutral markers were involved, the most probable *K* was changed to *K* = 2 (mean LnP(K) = −4,946.13; Δ*K* = 71.640) (Fig. 2). In the case of *K* = 2, the proportion of the first cluster was 88.3% in the wild population but 6.6 and 1.0% in the broodstock and offspring populations, respectively. In the case of neutral markers, these values changed to 87.8%, 6.3% and 1.5% in the wild, broodstock and offspring populations, respectively. If *K* = 3, the proportion of the three clusters are 86.5%, 4.5% and 9% in the wild; 5%, 37% and 58% in the broodstock; and 9%, 63.8%, 35.2% in the offspring population. Using only neutral markers, the values change to 85.6%, 3% and 11.4% in the wild; 4.5%, 34.8% and 60.7% in the broodstock; and 1%, 35% and 64% in the offspring population.

DAPC analysis grouped the samples into three groups according to their origin, supporting the presence of one more distant (wild) and two partly overlapping (broodstock and offspring) groups. There was no difference between the displayed plots of DAPC based on all (neutral and outlier) or only on the neutral markers (Figs. 1 and 2).

**Figure 3:**
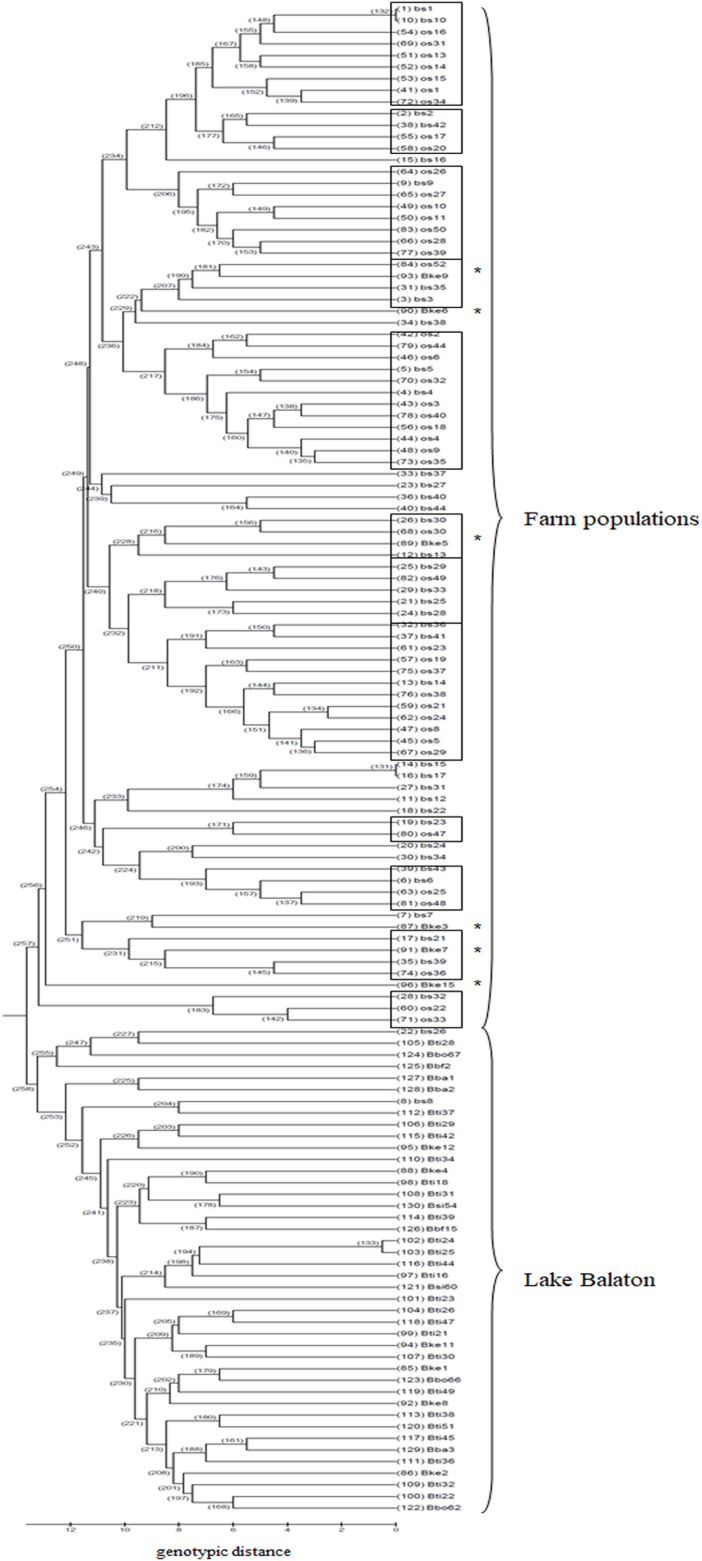
UPGMA tree for the 130 individuals calculated from genotypic distances. Individuals marked with * are from Lake Balaton. Abbreviations for individuals: bs, broodstock; os, offspring; Bke, Lake Balaton Keszthely; Bti, Lake Balaton-Tihany; Bbo, Lake Balaton-Boglár; Bbf, Lake Balaton-Balatonfüred; Bba, Lake Balaton-Balatonakali; Bsi, Lake Balaton-Siófok.

Based on the UPGMA tree (Fig. 3) the offspring population can be clustered into 12 families together with broodstock individuals. The 33 of the 44 offspring individuals were related with four of the broodstock pairs. The wild population shows separation, although six individuals from the Keszthely subpopulation were located in the groups containing farmed individuals based on genetic distances.

### Genetic selection

Three of the 14 loci were assigned as outliers in at least one of the methods, with the different methods identifying one, one and two outliers for LOSITAN, BayeScan and lnRH, respectively (Table 3). The other 13 markers were found to be neutral. However, only one of the outliers (MSL-9) was shared with at least two of the methodologically different outlier detection methods (Table 3).

**Table 3:** Outlier loci identified by LOSITAN, BayeScan, and lnRH. Column headings: LOSITAN: Het, expected heterozigozity; Fst, Fst-value; Prob, Probability; BayeScan: Prob, posterior probability for the model including selection; Log10 (PO), logarithm of Posterior Odds; *q*-value, *q*-value for the model including selection; LnRH: computed for population pairs Br/Off-Broodstock and Offspring; W/Br, Wild and Broodstock; W/Off, Wild and Offspring; Loci marked with * were identified as outlier.

Locus	Lositan			BayeScan			LnRH		
	Het	Fst	*P*	Prob	Log10 (PO)	*q*-value	Br/Off	W/Br	W/Off
Msl-1	0.77	0.133	0.972	0.09	−0.96	0.67	1.83	1.67	2.35*
Msl-3	0.69	0.098	0.844	0.08	−1.02	0.69	−1.28	0.91	0.34
Msl-5	0.91	0.051	0.823	0.14	−0.76	0.61	−0.55	0.67	0.41
Msl-6	0.80	0.022	0.277	0.58	0.14	0.22	−0.45	0.24	0.04
Msl-9	0.81	0.158	0.997*	0.04	−1.34	0.75	0.72	−2.07*	−1.67
Msl-2	0.72	0.068	0.736	0.12	−0.82	0.64	−0.87	0.67	0.27
Svi-18	0.79	0.019	0.248	0.22	−0.53	0.46	−0.03	0.77	0.72
Svi-4	0.77	0.110	0.946	0.03	−1.40	0.77	0.63	−1.45	−1.12
Svi-6	0.83	0.061	0.743	0.03	−1.40	0.78	−0.81	−0.36	−0.68
Svil-L7	0.63	0.008	0.188	0.97	1.52*	0.02	−1.18	−0.23	−0.71
PflaL3	0.66	0.058	0.674	0.05	−1.21	0.72	−0.60	−1.01	−1.21
SviL8	0.84	0.126	0.981	0.36	−0.23	0.36	0.56	0.65	0.85
Za138	0.86	0.097	0.966	0.22	−0.53	0.52	1.95	−0.76	0.08
Za199	0.51	0.018	0.270	0.05	−1.27	0.73	0.44	0.43	0.60

**Figure 4:**
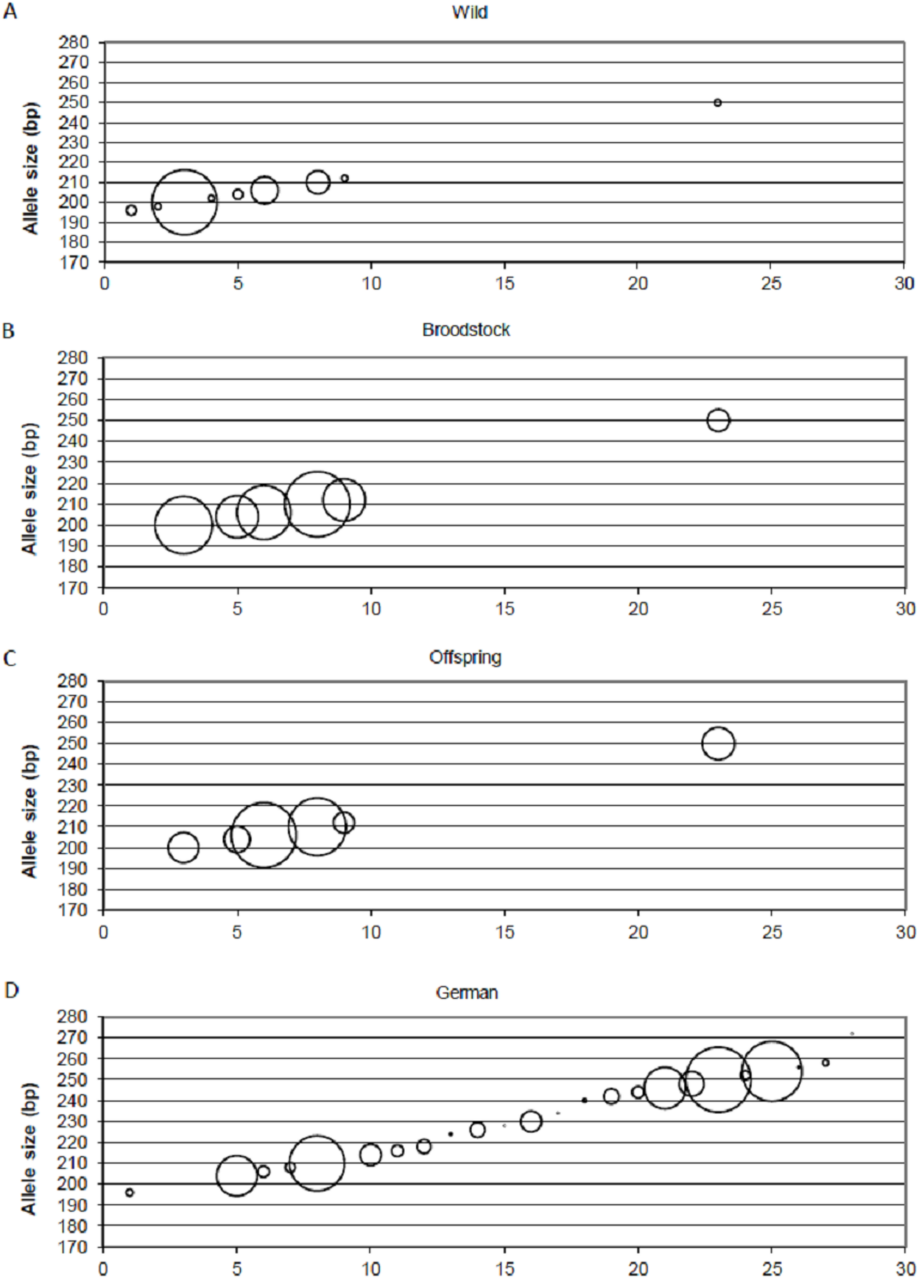
Changes in the allele frequencies of MSL-9 locus in the three populations. (A) Wild population. (B) Broodstock population. (C) Offspring population. (D) German population. The allele frequency of MSL-9 in the German population was calculated from the dataset by Eschbach et al. (2014). The size of the circles belonging to the individual alleles represents the frequency of the alleles. The *x* axes represent the individual alleles.

Positive selection resulted in the loss of small-sized alleles in the case of MSL-9 (Fig. 4). To identify the role of loci under selection in the genetic separation of cultured populations, the Fst values were recalculated using putative neutral and putative outlier loci separately. Excluding the outlier marker during the calculation of Fst values resulted in slightly reduced values in all population pairs (0.083, 0.111 and 0.009 between wild-broodstock, wild-offspring and broodstock-offspring population pairs). The results of AMOVA showed that only 7.13% of the total variance was found among populations. When using only the outlier locus in the calculation of population differentiation, a marked effect was detectable. In this case, the AMOVA showed that 15.83% of the estimated variance was among populations and the Fst values increased significantly in all population pairs (0.168, 0.269 and 0.030 between wild-broodstock, wild-offspring and broodstock-offspring population pairs), indicating that this marker is responsible for the level of genetic differentiation.

The putative outlier marker sequence MSL-9 was (250 bp) BLAST to two perch transcriptomes. The sequence showed homology with two transcribed perch RNA sequences (Accession: GFIQ01024864.1 and GFIQ01024865.1; Query cover 70%, Expect value: 9e^−19^; Percent of Identity: 98.36%). The further blast of these perch sequences to databases of other species resulted in an association of the sequence with *Perca flavescens* transthyretin precursor mRNA (Accession: HQ206530.1; *E* value: 0.0; Per. Ident: 98.95%).

## Discussion

Supporting our expectations, the reduction of genetic diversity during the pond culture was detectable in the average allele number, allelic richness and private allelic richness data. However, heterozygosity measures were similar across all groups. Despite the reduction, both the pond cultured and wild populations showed high genetic variability. Our set of microsatellite markers overlapped with markers applied in other studies (four to eight markers were identical) in all but one of the cases where literature was concerned with information on the genetic diversity of pikeperch populations. The heterozygosity levels and allelic richness showed a wide range: Finnish populations He = 0.3–0.46, Ar = 3.3–5.5 (Säisä et al., 2010) and He = 0.51, Ar = 3.6–4.6 (Salminen et al., 2012); Fennoscandian region, Ar = 2.8–8 (Björklund, Aho & Larsson, 2007) Caspian Sea He = 0.50–0.54 (Gharibkhani et al., 2009); Rhone delta He = 0.64–0.74, Ar = 4.0–6.0 (Poulet et al., 2009); Uzbek populations He = 0.74 (Khurshut & Kohlmann, 2009) and lower Volga Basin He = 0.79–0.82, Ar = 9.57–10.64 (Kusishchin et al., 2018). Although our examined region was the Danube river basin, the genetic variability of the stocks was higher than the German populations (He = 0.62 and Ar = 4.5) (Eschbach et al., 2014), the wild population showed similar values to the Volga populations.

In our study the genetic diversity of the wild population was higher than in both the farm broodstock and offspring populations. The allele number and the allelic richness of the offspring population was almost half that of the wild population. Khadher et al. (2016) reported that farm populations of Eurasian perch (*Perca fluviatilis*) had higher genetic diversity compared to the source population, contrary to our results. Moreover, the offspring populations had similar allelic richness and heterozygosity compared to the broodstock and only the allele number decreased with 10–21% in the offspring. Data on the genetic variability of Hungarian wild and farmed pikeperch populations has been published in only two studies. Tsaparis et al. (2015) compared the genetic diversity of 12 domesticated populations (from six countries including Hungary) and a Hungarian wild population using 12 microsatellites. The mean number of alleles and the level of heterozygosity (He) were the second highest in the Hungarian farm population (Na = 7.55; He = 0.71) which exceeded the value of the wild population (Na = 6.00; He = 0.67). Kánainé Sipos et al. (2019) estimated the genetic diversity of 10 pikeperch populations in the Danube basin (including Balaton and Attala populations) using a different microsatellite set. The genetic diversity of the two populations was moderate (Ar = 3.49 and 3.50 He = 0.46 and 0.52 in the Balaton and Attala population, respectively) compared to the other populations (Ar = 2.48–3.97 He = 0.48–0.59), but was lower than in the present study.

In spite of its higher diversity, the wild population showed the signs of a recent bottleneck event. Specziár & Turcsányi (2017) reported that the pikeperch population of Lake Balaton is characterized by a high rate of harvesting, an early-life dietary bottleneck and a high rate of predation and cannibalism-related juvenile mortality. All of these factors can greatly reduce population size, resulting in changes in the genetic structure.

The genetic separation of the farmed and the wild populations is already evident with neutral markers. However, DAPC analyses or the involvement of outlier locus in SRUCTURE analysis resulted in further separation of the offspring population from the broodstock. Effective population size decreased in both farm populations and offspring also showed a significant reduction compared to the broodstock. An unequal family contribution was observed within the offspring population (Table S6). The 75% of the offspring population was clustered in 4 families (Fig. 3). Khadher et al. (2016) examined the relationship of farmed Eurasian perch populations that likely shared common origin of Lake Geneva. They found family structuring with an unequal family contribution among broodstocks and their offspring and the effective population size decreased in all offspring groups. Aho et al. (2006) described a negative correlation between the current population size and the years since founding in the hatchery populations of brown trout.

Broodstock population size was estimated as Ne = 67.9, which agrees with the known number of animals. However, the Ne of the offspring populations was extremely low, with 15.1 individuals (according to the LD method) or 31.3 individuals (temporal method). Christie et al. (2012b) found similar results when examining the effective population size of hatchery broodstocks of steelhead trout (*Oncorhynchus mykiss*) involved in a supplementation program in the Hood River population. Despite a total of 40–80 fish being used as broodstock each year, the Ne of the parents estimated from the offspring generation was small (average 24.9, ranging from 16.5 to 36.7). The low population size caused decreased allelic richness, increased average relatedness and substantial levels of genetic drift in hatchery fish.

In the present study, the decrease in genetic diversity in the farmed populations coupled with the decrease of effective population sizes suggested a strong founder effect in broodstock. This effect was more intense in the offspring population, which could be caused by several factors. The most probable is the variance in reproductive success of fish resulting in different hatching rates of eggs and/or different survival ratios of fish larvae (as a consequence of varying quality of the eggs). Other factors could be the effect of age or unbalanced sex ratio, although the effect of these factors is limited by the selection before controlled propagation. Araki & Schmid (2010) illustrated in their review that about one-third of the 70 studies comparing hatchery and wild stocks showed significantly negative effects of hatchery rearing on the fitness of stocked fish and reduced genetic variation in hatchery populations.

In addition to the reduction of Ne and genetic diversity, a significant deviation from HWE was observed in four of the 14 loci in the offspring population. Although heterozygosity was high, the F_IS_ value was negative in three of the loci (MSL-5, MSL-6 and Svi18) suggesting that the founder effect or genetic drift significantly affected the offspring population. In the case of MSL-1, one of the outlier methods (LnRH) suggested selection on the locus.

Beyond the founder effect and genetic drift, the source of genetic differentiation could be the selection of different environmental factors. In the present study, three of the 15 loci were detected as an outlier using one of the selection detection methods and one of them was supported by at least two of the three independent methods. DAPC supports the separation of the three populations independently from the inclusion of this outlier locus, but structure analysis confirms the further separation of the offspring population as an effect of this locus (MSL-9). The comparison between allele frequencies of MSL-9 in the present study and in German populations (with a range between 196 and 254 bases pairs in the dataset of Eschbach et al. (2014)) (Fig. 4) shows that the Hungarian wild population has a narrowed allele range containing small-sized alleles. Pond culture moves this range toward the larger allele sizes, which are more common in German native populations, but it is not possible to distinguish among variance in reproductive success and family-correlated survival. As the LNRH statistic could not prove the existence of selection between wild and offspring populations, further investigation is needed to assess the potential impact of selection. Christie et al. (2016) demonstrated that “domestication” already occurs in the first generation of hatchery rearing in steelhead trout (*Oncorhynchus mykiss*). The process influences the expression of more than 700 genes involved in the adaptation to the culture environment due to the modification of the immune response or metabolism. Similar results were described by Waters et al. (2015).

Nevertheless, selection has a minor effect compared to the genetic effect of low effective population sizes. Stocking with offspring populations could pose significant genetic risks to the wild population. One of the main sources of genetic change originates in the “Ryman–Laikre” effect created by reduced effective population size of the hatchery stock compared to the original wild population. It is most aggressive if the hatchery broodstock is small, if the proportion of the released offspring is high and if the RRS of the released fish is high (Christie et al., 2012b).

According to the Ryman–Laikre model, 62.5 and 15.1 broodstock Ne assumes reproductive success of about 11.5% and 2.5%, respectively to maintain the size of the 1,032 population. Although RSS is not known in Lake Balaton, Salminen et al. (2012) reported on presumably highly variable reproductive success in pikeperch. Reproductive success above 11.5% combined with the calculated 15.1 Ne of the offspring population may already cause a significant (approximately 40%) decrease in the population effective size of the captive-wild system. Waples et al. (2016) pointed out that, in addition to the proportion of reproductive captive individuals, the ratio of Ne/N in captive and wild stocks (β) is also a key factor in success of supplementation programs. Stocking large number of individuals with a low Ne results in a low β value. The supplementation program can only increase overall Ne if β > 1. The importance of β increases as the proportion of reproductive captive individuals within the stock increases. Specziár & Turcsányi (2017) reported on the annual stocking of 1–1.5 million fingerlings into Lake Balaton. Based on mark–recapture data, they found an average 7.7% survival at the size category <150 g and a rate of 30.1% if fingerling above 300 g weight were stocked. This amounts to a total of 115–300 thousand surviving individuals per year. As pikeperch is a non-migratory fish (with maximum distances of spawning movements less than 35 km, Lappalainen, Dörner & Wysujack, 2003), local populations should be carefully stocked according to the Ne/N ratio of hatchery stock.

## Conclusions

Our case study shows that the lower population size of the broodstock resulted in a reduced, but still high, genetic variability compared to that of the natural population. This variability and population size appears to be sufficient for the aquaculture production of this species, but there is still a genetic risk in supplementation of natural waters. The same parameters in the offspring population were significantly reduced. When using these individuals for both aquaculture and stock enhancement purposes, the genetic decline of the affected populations is expected. Altogether, the present study provides support for the reassessment of the suitability of pond cultured stocks in the supportive breeding of natural fish populations and it highlights the importance of the genetic monitoring of both farmed and natural populations.

## Supplemental Information

10.7717/peerj.8745/supp-1 Supplemental Information 1 Raw data.

10.7717/peerj.8745/supp-2 Supplemental Information 2 The final reaction conditions and temperature profiles of the multiplex reactions.

10.7717/peerj.8745/supp-3 Supplemental Information 3 Summary statistics for each locus.

10.7717/peerj.8745/supp-4 Supplemental Information 4 Table of genetic divergence by locus.

10.7717/peerj.8745/supp-5 Supplemental Information 5 Table of allele frequencies by locus and by population.

10.7717/peerj.8745/supp-6 Supplemental Information 6 Matrix of maximum likelihood relatedness.

## Additional Information and Declarations

### Field Study Permissions

The following information was supplied relating to field study approvals (i.e., approving body and any reference numbers): Field experiments were approved by the Hungarian Ministry of Agriculture (permit number: HHgF/269-1/2015)

### Data Availability

The following information was supplied regarding data availability: The raw data measurements are available as a Supplemental File.
