# Supplementary material for: Genetic consequences of pond production of a pikeperch (Sander lucioperca L.) stock with natural origin: the effects of changed selection pressure and reduced population size
Source: PeerJ. 2020 Mar 17;8:e8745. doi: 10.7717/peerj.8745 (PMC7083162; doi:10.7717/peerj.8745)
Supplement: Supplemental Information 2 [file peerj-08-8745-s002.doc]

| **A reaction** | |
| --- | --- |
| PCR buffer | 1x |
| MgCl2 | 1.5 mM |
| dNTP | 200 µM |
| MSL 1 forward and reverse primer | 0.1 µM – 0.1 µM |
| MSL 3 forward and reverse primer | 0.066 µM - 0.066 µM |
| MSL 5 forward and reverse primer | 0.266 µM – 0.266 µM |
| MSL 6 forward and reverse primer | 0.2 µM – 0.2 µM |
| MSL 9 forward and reverse primer | 0.2 µM – 0.2 µM |
| Taq DNA polymerase | 1.2 U/µl |
| genomic DNA template | 50 ng |
| **B reaction** | |
| PCR buffer | 1x |
| MgCl2 | 1.5 mM |
| dNTP | 200 µM |
| MSL 2 forward and reverse primer | 0.2 µM – 0.2 µM |
| Svi-4 forward and reverse primer | 0.1 µM – 0.1 µM |
| Svi-6 forward and reverse primer | 0.1 µM – 0.1 µM |
| Svi-L7 forward and reverse primer | 0.2 µM – 0.2 µM |
| Svi-18 forward and reverse primer | 0.2 µM – 0.2 µM |
| Pfla-L8 forward and reverse primer | 0.2 µM – 0.2 µM |
| Taq DNA polymerase | 1.2 U/µl |
| genomic DNA template | 50 ng |
| **C reaction** | |
| PCR buffer | 1x |
| MgCl2 | 1.5 mM |
| dNTP | 200 µM |
| Pfla3 forward and reverse primer | 0.1 µM – 0.1 µM |
| Za138 forward and reverse primer | 0.25 µM - 0.25 µM |
| Za199 forward and reverse primer | 0.05 µM – 0.05 µM |
| Svi-L8 forward and reverse primer | 0.3 µM – 0.3 µM |
| Taq DNA polymerase | 1.2 U/µl |
| genomic DNA template | 50 ng |

The temperature profiles for multiplex A and B amplifications were: 10 min at 94°C initial denaturation, 35 cycles for 60 s at 95°C, 90 s at annealing temperature (56 °C in case of multiplex A and 55°C in case of multiplex B), and 60 s at 72°C, followed by an extension at 72°C for 20 min. The temperature profile for the multiplex C amplification was: 10 min at 94°C initial denaturation, five cycles for 60 s at 95°C, 90 s at 50 °C, and 60 s at 72°C, 30 cycles for 60 s at 95°C, 90 s at 47 °C, and 60 s at 72°C, followed by an extension at 72°C for 20 min. The PCR products were kept at 4°C
